# Passive data do not improve prediction or detection of alcohol consumption beyond temporal patterns in major depression: A 90-day cross-validated study

**DOI:** 10.1016/j.addbeh.2026.108624

**Published:** 2026-01-26

**Authors:** Anna M. Langener, Dawson Haddox, Daniel M. Mackin, George D. Price, Damien Lekkas, Amanda C. Collins, Tess Z. Griffin, Michael V. Heinz, Matthew D. Nemesure, Subigya Nepal, Arvind Pillai, Andrew T. Campbell, Nicholas C. Jacobson

**Affiliations:** a Center for Technology and Behavioral Health, Geisel School of Medicine, Dartmouth College, Lebanon, NH, United States; b Department of Psychology, University of Arizona, Tucson, AZ, United States; c Department of Psychiatry, Geisel School of Medicine, Dartmouth College, Lebanon, NH, United States; d Quantitative Biomedical Sciences Program, Dartmouth College, Lebanon, NH, United States; e Department of Medical Social Sciences, Feinberg School of Medicine, Northwestern University, Chicago, IL, United States; f Deparment of Psychiatry, Massachusetts General Hospital, Boston, MA, United States; g Department of Psychiatry, Harvard Medical School, Boston, MA, United States; h Digital Data Design Institute, Harvard Business School, Harvard University, Cambridge, MA, United States; i Stanford Institute for Human-Centered-AI, Stanford University, Palo Alto, CA, United States; j Department of Computer Science, Dartmouth College, Lebanon, NH, United States; k Department of Biomedical Data Science, Geisel School of Medicine, Dartmouth College, Lebanon, NH, United States

**Keywords:** Major depressive disorder, Passive sensing, Deep learning, Meta learner, Alcohol use, Just-in-time adaptive interventions, JITAI

## Abstract

Major Depressive Disorder (MDD) is one of the most prevalent psychological disorders and frequently co-occurs with alcohol use disorders, increasing the risk of functional impairment. Monitoring alcohol use during depression treatment is therefore critical for early intervention. Passively collected data via devices like smartphones and smartwatches, offers a low-burden method for monitoring behavior in real time. This study investigated whether deep learning models trained on passively collected data (i.e., accelerometer, heart rate, respiratory rate, screen usage, and GPS data) could detect and predict alcohol use in individuals with MDD. Data were collected from 300 clinically depressed individuals who were enrolled in the Tracking Depression Study, a 90-day longitudinal study. Participants self-reported their alcohol use every week by completing the Timeline FollowBack. We trained models to predict same-day and next-day alcohol use. To validate these models, we split the data by participant, so that predictions were made on individuals who were not included in the training set. The models achieved moderate performance (mean AUC = 0.67 for both prediction tasks) when capturing both interindividual (between-person) and intraindividual (within-person) variability. Similar performances were observed when evaluating the model exclusively on predicting intraindividual variability (AUCs = 0.69 same-day, 0.68 next-day). However, model performance remained comparable to a baseline using only the day of week as predictor. These findings suggest that much of the predictive signal derives from temporal patterns. This indicates that interventions aligned with such temporal cues may already be effective, and that the added value of our model appears limited.

## 1. Introduction

Major Depressive Disorder (MDD) is one of the most prevalent psychological disorders worldwide and serves as a leading cause of disability (Mojtabai et al., 2016; Reddy, 2010). Individuals suffering from MDD often exhibit symptoms such as persistent low mood, sleep disturbances, a lack of motivation and energy, reduced appetite, and suicidal thoughts (Fava & Kendler, 2000). Additionally, previous research has established an association between MDD and substance use disorders, such as alcohol dependencies (e.g., Hunt et al., 2020; Sullivan et al., 2005). For example, between 1990 and 2019, 20.8% of individuals with MDD also meet criteria for alcohol use disorder (Hunt et al., 2020). While non-clinical occasional alcohol use may not directly influence the course of MDD (Schouten et al., 2023), the comorbidity of clinically relevant substance use can lead to heightened levels of social and personal impairment, along with a heightened vulnerability to other psychiatric conditions (Davis et al., 2008). Consequently, monitoring alcohol use and initiating early intervention when alcohol use emerges can be beneficial in the context of depression treatment (Currie et al., 2005).

To monitor alcohol use closely and enable timely interventions, methods are needed that can capture alcohol use on a granular time scale with a low participant burden. Such a granular time scale can, for example, be beneficial to help identify potential moments to deliver direct interventions if alcohol use occurs, such as with just-in-time adaptive interventions (JITAIs). These interventions are delivered in real-time during individuals’ daily lives, offering a dynamic approach to addressing mental health concerns (Nahum-Shani et al., 2017). Effective implementation of JITAIs requires identifying specific time windows that trigger the intervention. Up to now, JITAIs targeting alcohol and other substance use reduction have often relied on static rules derived from questionnaires or location data to determine when to deliver an intervention (Perski et al., 2022). However, these static rules are limited because they fail to account for within-person variability and the dynamic, context-dependent nature of behavior in everyday life. Additionally, relying on questionnaires can be burdensome for participants. Enhancing our ability to accurately detect same-day alcohol use and predict next-day alcohol use could increase the effectiveness of JITAIs, potentially reducing the quantity of alcohol use and its associated negative consequences, or preventing consumption from occurring in the first place (Coughlin et al., 2021).

Previous research has demonstrated the potential of predicting alcohol consumption using both active and passive data sources. For instance, in a recent study, researchers employed participants’ responses to daily questionnaires regarding stress and hopefulness, along with factors such as the day of the week and sex, to forecast the next day’s drinking behavior (Coughlin et al., 2021). This approach yielded an area under the curve (AUC) of 0.76 in a sample of 51 emerging adults, demonstrating its effectiveness in predicting such behavior (Coughlin et al., 2021). However, reliance on daily questionnaires may lead to participant fatigue over time, limiting long-term feasibility.

To address these limitations, recent efforts have focused on leveraging only passively collected data, which can reduce participant burden while enabling continuous and unobtrusive monitoring (Campbell et al., 2008). Devices such as smartphones and smartwatches can be used to collect data passively in real time (Onnela, 2021). For example, data on heart rate, GPS, screen time, movement, and respiratory rate is frequently gathered. Passive sensing approaches, often referred to as digital phenotyping (Torous et al., 2016), allow for real-time behavioral monitoring at scale (Luhmann, 2017; Onnela, 2021). When combined with machine learning techniques, such data streams may support same-day alcohol use detection, behavioral prediction, and the identification of optimal intervention moments.

One promising example is a study by Bae et al. (2023), which predicted imminent same-day binge drinking with 95% accuracy on weekends and 94.3% accuracy on weekdays using only passive data from young adults. A strength of this study is that it predicted alcohol use prior to its occurrence (i.e., forecasting in the future), using data collected up to one hour before reported drinking, and evaluated model performance across different lookback windows ranging from 1 to 6 h. While these results are impressive, the study used a cross-validation strategy that split data by time points rather than by individuals, potentially inflating performance estimates and limiting the model’s generalizability to new users (Langener & Jacobson, 2025; Saeb et al., 2017). This matters, because individuals often differ from each other and the model might have only learned patterns that are specific to certain participants. Similarly, the model may have learned to distinguish between high- and low-frequency drinkers rather than detecting specific moments in which someone was drinking. Since the reported results aggregated both intra- and inter-individual variability, it’s unclear whether the model can accurately predict drinking behavior within the same individual in everyday life.

Building on this line of work, the current study aims to evaluate the effectiveness of deep learning models in detecting same-day and predicting next-day alcohol consumption in real time among individuals diagnosed with MDD. Here, “same-day” refers to detecting alcohol use on the day it occurs (meaning that features may include data collected before, during, and after the drinking episode), whereas “next-day” refers to predicting alcohol use on the following day using data collected prior to that day. We focus exclusively on passive data collected from smartphones and smartwatches to reduce participant burden and use a cross-validation strategy that tests model generalizability across individuals. Specifically, we investigate whether these passively collected measures can accurately detect same-day alcohol consumption and predict next-day use.

## 2. Methods

### 2.1. Sample

Data were drawn from the Tracking Depression Study, an R01 project funded by the National Institute of Mental Health (NIMH) and the National Institute of General Medical Sciences (Jacobson, 2021). Participants, enrolled for 90 days, were recruited in the US via online ads (Instagram, Facebook, Google). The study was approved by Dartmouth’s IRB (STUDY00032081) and conducted in accordance with the Declaration of Helsinki (for more information on the study see Collins et al., 2024; Langener et al., 2025). Written and verbal informed consent was obtained per approved procedures. Eligibility required participants to be 18+, diagnosed with MDD, and Android users. Exclusion criteria included acute suicidal risk, bipolar disorder, psychotic symptoms, or not meeting MDD criteria in the past 30 days. This was assessed using a Structured Clinical Interview for DSM-5 (SCID) administered via Zoom by either a psychiatrist or a postdoctoral research fellow in clinical psychology. Of the 431 participants interviewed, 312 met criteria for a current major depressive disorder (MDD) episode and did not report a history of (i) mania, (ii) psychosis, or (iii) moderate to high acute suicide risk; these participants were invited to take part in the main study.

The final sample consisted of 300 individuals, who were primarily White (79%), heterosexual (66%), and female (84%), with an average age of 40 years. The racial and ethnic diversity of the sample, which included Black, Asian, and American Indian or Alaska Native participants, closely mirrored the U.S. population of individuals with MDD, while Hispanic and Latino participants (12%) were also represented in a similar proportion. Most participants (93%) had some college education, with 68% holding a college degree, and more than half were employed (61%).

**Fig. 1.**
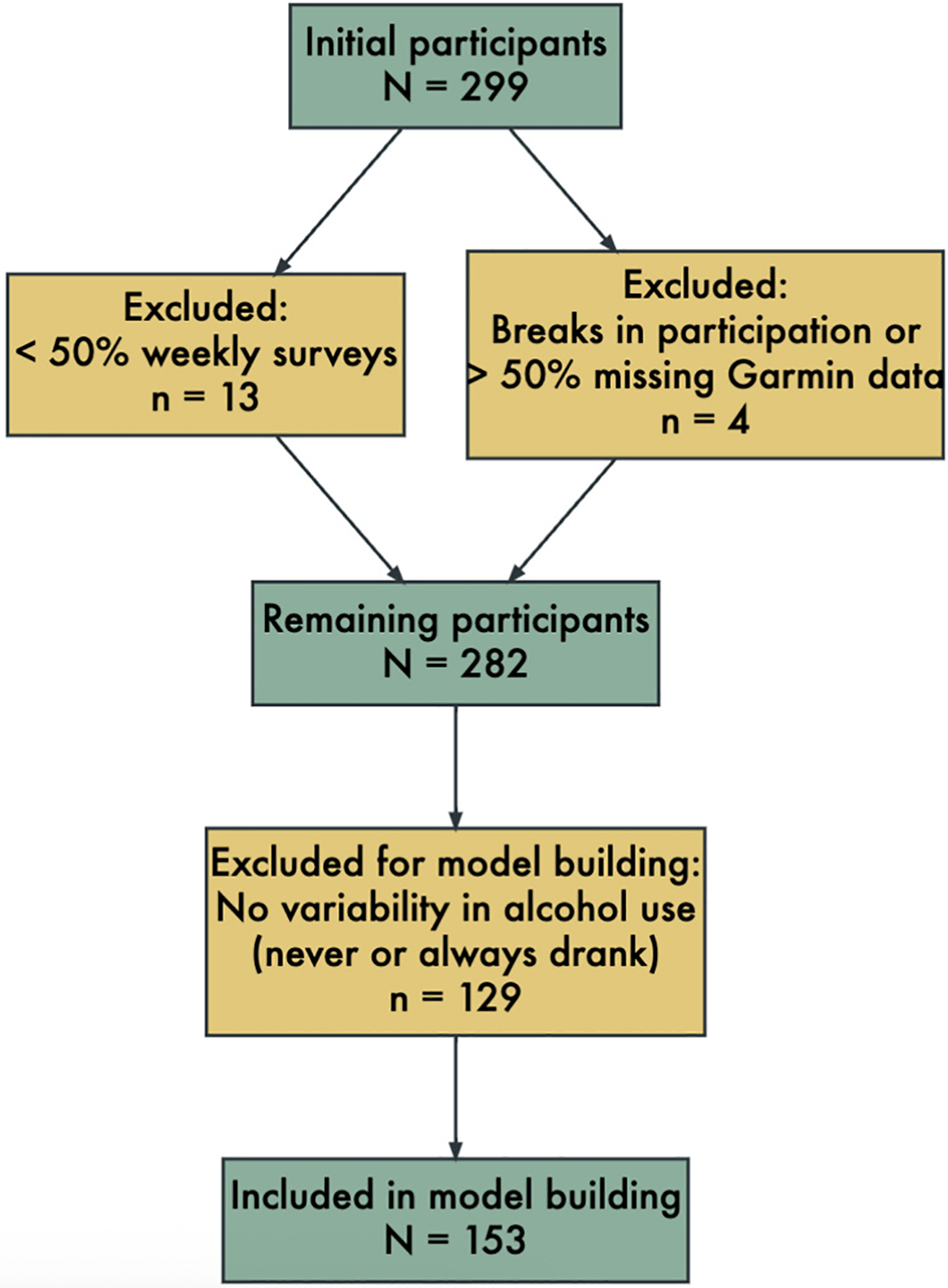
Participant Flowchart.

Participants who completed less than 50% of weekly surveys or wore the Garmin watch for under 50% of the study were excluded. Of the initial 299 participants,^1^ 13 were removed for completing fewer (see Fig. 1 for an overview). Another four participants were excluded due to breaks in participation or missing more than 50% of Garmin sensor data, leaving 282. For model building, only those with variability in alcohol use were included, excluding individuals who never or always drank (N = 153). This decision was driven by the intended use case of our models (JITAI), where only individuals with fluctuations in alcohol use would benefit from such interventions. Additionally, we aimed to prevent the model from merely distinguishing between users and non-users, focusing instead on identifying optimal moments for intervention delivery.

A summary of participant demographics is presented in Table 1. The analyzed sample comprised 153 participants with a mean age of 37.9 years (range: 19–78). Of these individuals, 8 met criteria for past-year alcohol use disorder (AUD), 42 met criteria for lifetime AUD, and 3 met criteria for subthreshold AUD. Comorbid psychiatric diagnoses are detailed in Supplementary Table 1. The most prevalent comorbid condition was lifetime generalized anxiety disorder, which was present in 63.4% of participants. Overall, the demographic composition of the sample closely mirrors that of the U.S. population of individuals with major depressive disorder..

### 2.2. Procedure

Once enrolled, participants were instructed to install the MLife smartphone application on their Android devices (R. Wang et al., 2014). This app passively collected various data, including location, activity, battery status, SMS logs, and call logs. In addition to passive data collection, participants were prompted to complete daily questionnaires, though the data from these surveys were not used in the current analysis. Weekly surveys were also administered, where participants reported on the days they consumed alcohol or used other substances during the previous week.

Participants were provided with a Garmin smartwatch (Vivoactive 4 s) and instructed to wear it throughout the study period. This device tracked various physiological data, such as heart rate, heart rate variability, oxygen saturation, and steps.

Before the study began, each participant received a comprehensive explanation of the study during an onboarding session. To promote engagement, participants received biweekly feedback regarding their participation, including details on completed surveys and smartwatch usage. Weekly reminders were sent to encourage the completion of substance use surveys. Upon completion of the study, participants received feedback on their symptoms over the 90-day period. In total, participants could earn rewards up to $487, based on their completion of all study components.

### 2.3. Measures

#### 2.3.1. Outcome metrics: Alcohol consumption

**Fig. 2.**
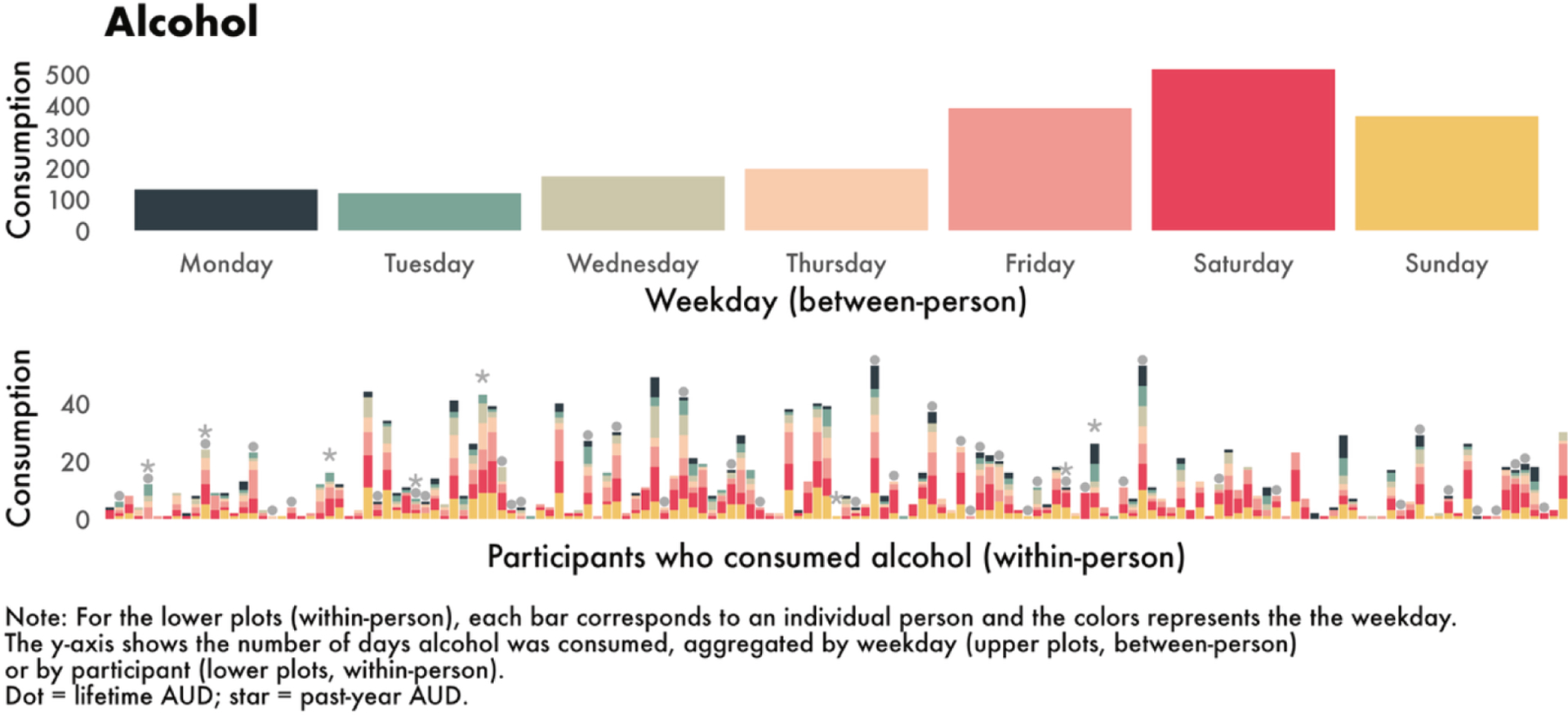
Overview of alcohol consumption.

Each week, participants completed the Timeline FollowBack to report on the days of the previous week that they consumed alcohol (Robinson et al., 2014). For more information on the measures see also Collins et al. (2024). From these weekly reports, we derived daily variables indicating alcohol consumption (binary: yes/no).^2^ For alcohol use, 153 participants exhibited variability in their consumption (i.e., they did not consume alcohol every day or abstain entirely, see 2). Among the 153 individuals, 8 met the threshold for past-year AUD (indicated by a star in Fig. 2), 42 met the threshold for lifetime AUD (indicated by a dot in Fig. 2). On average, these participants had 79.9 days of data (median = 82, SD = 9.2, min = 45, max = 89). Note that if a weekly questionnaire was missing, the entire prior week was coded as missing and that we excluded participants that had more than 50% of missing questionnaires. On average, these participants consumed alcohol on 12.4 days (median = 8, SD = 12.4, min = 1, max = 53).

#### 2.3.2. Predictors: Passive sensing data

In our final model, we included six predictors: zero-crossing rate (ZCR), heart rate, respiratory rate, screen usage, visited places, distance from a cannabis dispensary, and weekday.

ZCR, heart rate, respiratory rate, and screen usage were included because prior research has identified movement and device usage as key indicators for predicting substance use (Bae et al., 2021, 2023). Additionally, moderate alcohol use, such as two drinks of wine, is linked to an increased heart rate (Spaak et al., 2008). We included distance from a cannabis dispensary because certain neighborhood characteristics may serve as a meaningful predictor of alcohol use and can influence drinking behavior (Epstein et al., 2020; Kirchner & Shiffman, 2016). Lastly, day of the week was incorporated as previous studies have found it to be a strong predictor of substance use (Bae et al., 2021, 2023; Collins et al., 2024). In the following, we provide a detailed explanation of each variable (see Table 2 for an overview). For further details, including the code used to generate these variables and a README file with additional information, see https://osf.io/wseyd/.

ZCR measures how often a signal crosses zero, making it an indicator of movement and activity frequency. This signal is derived from accelerometer data (Garmin Health, 2023) and was recorded every 30 s. For use in our deep learning model, ZCR was summarized into 60-second intervals by calculating the mean. Following a previously published approach, outliers were handled by removing values that exceeded 722 zero crossings (we used a similar approach in Langener et al., 2025).

Heart rate was recorded every 10 s. Similar to ZCR, we summarized heart rate into 60-second intervals by calculating the mean. To exclude outliers, we estimated the maximum heart rate using the formula 220 minus age (Nes et al., 2013). Since participants were required to be at least 18 years old, we excluded values above 202. For the minimum heart rate, we applied a threshold of 27 bpm, based on the lowest recorded heart rate in male elite runners during sleep (Jensen-Urstad et al., 1997).

Respiratory rate was recorded every minute. In our dataset, the minimum recorded value was five breaths per minute. We chose not to set a lower threshold, as slow breathing (e.g., 5.5 breaths per minute) is commonly used in meditation exercises, which we aimed to retain (Lin et al., 2014), However, we excluded values exceeding 60 breaths per minute, as this is the maximum observed in well-trained athletes during intense exercise (Nicolò et al., 2016).

Screen usage was measured based on whether the screen was turned on or off. To summarize this data into 60-second intervals, we calculated the sum, with a maximum value of 60 indicating continuous screen activity for the entire minute.

Visited places were identified using GPS data. We labeled these locations based on Google’s classification and further grouped them into six categories for this project, because we wanted to reduce dimensionality of the predictors and focus on those categories that are important to predict substance use. Thus, the first category includes places associated with alcohol and substance use, such as bars, nightclubs, and casinos. The second category consists solely of liquor stores, as they are key locations for predicting alcohol use. The third category encompasses other locations where participants might have purchased alcohol, including gas stations, retail stores, supermarkets, and shopping malls. The fourth category includes restaurants, while the fifth consists of sports-related places such as gyms and fitness centers. The final category covers travel- and vacation-related locations, such as hotels and resorts. Each category was represented as a binary variable, indicating whether the participant visited a place within that category.

Based on GPS data, we also calculated the minimum distance participants were from a cannabis dispensary on each day, as certain neighborhood characteristics can be related to alcohol use (Epstein et al., 2020; Kirchner & Shiffman, 2016). This was determined using a dispensary list we compiled across the US based on weedmaps.com. Lastly, each weekday was included as a dummy variable.

In our approach, we chose not to impute or remove missing sensor data throughout the day (i.e., accelerometer, heart rate, respiratory rate, and screen usage), as we recognized that these gaps might contain valuable insights. As a result, missing values were assigned a flag value of 0.01 (before the data was scaled). This value was selected because it falls outside the normal range of observed (unscaled) data. For example, the minimum recorded heart rate in our dataset was 23 bpm, and screen usage, measured as the number of seconds the screen was on per minute, ranged from 0 to 60 in increments of 1, making 0.01 a clearly distinguishable marker of missingness. For, GPS derived features (i.e., places visited and distance to cannabis dispensary), we opted for a mixed approach of handling missing values. For “visited place”, we followed the same flagging approach, using a value of −1, which falls outside the valid data range and therefore serves as an effective missingness marker. For the distance to the next cannabis dispensary, we employed a masking approach, excluding missing values during model training. This decision was made because missingness in this feature was not independent, but rather redundant with missingness in places visited, since both were derived from the same GPS sensor.

**Fig. 3.**
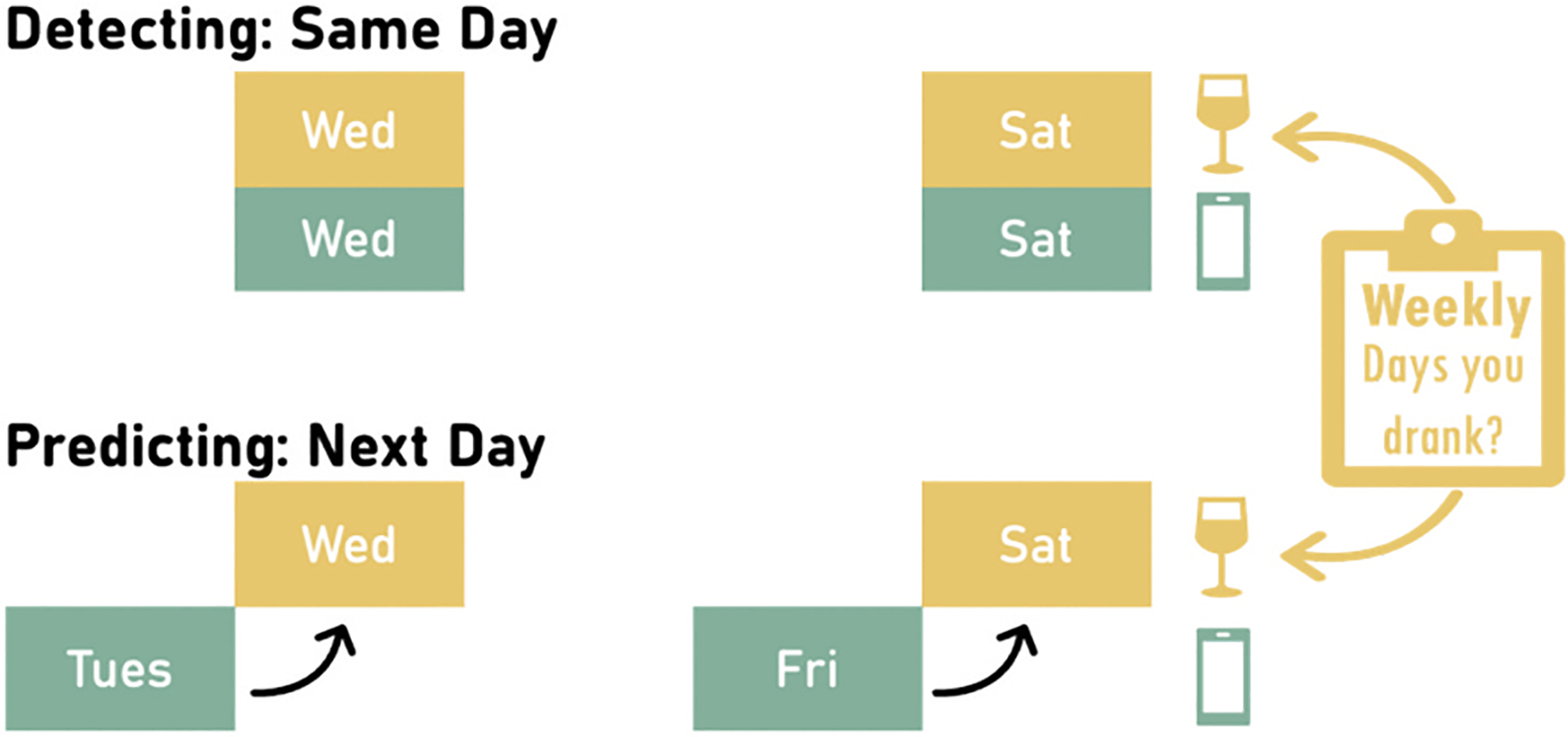
Temporal Relationship between outcome (alcohol consumption) and predictors (passive data).

Each variable was included on a daily timescale. For ZCR, heart rate, respiratory rate, and screen usage, we incorporated data for each minute of the day. To detect alcohol use, models incorporated passive sensor features aggregated from the *same* day on which alcohol consumption was reported. Because these features may include data collected before, during, and after the drinking episode, same-day models are interpreted as detecting alcohol use on that day rather than prospectively identifying imminent drinking. To predict alcohol use, models exclusively incorporated passive sensor features aggregated from the *preceding* day relative to the reported drinking day. This approach ensured that all predictor data temporally preceded the alcohol use outcome, thereby reducing the risk of information leakage and supporting a prospective interpretation (see Fig. 3). We chose a daily timescale because it aligned with the information we had regarding alcohol consumption based on the weekly Timeline FollowBack. Continuous variables were scaled within each training set before training, using a min–max transformation to, which has been shown to enhance model performance and efficiency (Shahriyari, 2019).

### 2.4. Prediction model

#### 2.4.1. Overview and meta learner

We explored a range of machine learning and deep learning architectures and evaluated their performance on validation sets (see Model Development in Supplementary Material). This section presents our final model: a stacked ensemble. In this approach, predictions from three diverse base learners were used as input features for a logistic regression *meta*-learner. The base learners included: (1) a multi-head, multi-channel deep learning model, (2) a Random Forest model, and (3) a Random Forest model trained without weekdays as a predictor. The multi-head, multi-channel deep learning model was selected iteratively, with its architecture treated as a hyperparameter. We selected Random Forests as base learners model due to its robustness against overfitting and its ability to handle multicollinearity among predictors. Random Forests are also well-suited for high-dimensional data and have been widely used in digital phenotyping studies for predicting mental health outcomes (Benoit et al., 2020; Breiman, 2001). To ensure the ensemble could capture patterns that are both dependent and independent of temporal structures, we ran the Random Forest model twice, once including the weekday as a predictor and once without it.

#### 2.4.2. Model 1) deep learning model

To integrate multiple predictors into a single framework, we employed a multi-head, multi-channel deep learning architecture (Canizo et al., 2019), where each head processes a different type of input. The model was built in Python (3.10) using *Tensorflow* (2.18.0) and *Keras* (3.8.0).

The final model consists of four distinct heads, each corresponding to a specific data source: (1) google maps features, (2) cannabis dispensary features, (3) weekday features, and (4) physiological and behavioral data (including heart rate, ZCR, respiratory rate, and screen activity). The latter four features were grouped into a single head because they shared a similar data structure, enabling efficient processing within the same framework. The first three heads use dense layers to learn meaningful patterns from individual sensor types, while the fourth head processes time-series physiological data by converting it into 2D images via Gramian Angular Fields and applying a 2D convolutional neural network architecture to capture temporal dependencies (Krizhevsky et al., 2017). After processing each data type separately, their outputs are concatenated and further processed through dense layers to learn combined relationships, producing a final output predicting the likelihood of alcohol consumption. For a full overview of the model and an explanation of the structure of each head please see Supplementary Material (Model Architecture), for the full code please see https://osf.io/wseyd/).

#### 2.4.3. Model 2) + Model 3) random forest

The remaining two base models were Random Forest classifiers. Both models shared an identical structure and feature set, with the only distinction being the inclusion of the weekday feature in one model and its exclusion in the other. For each model, we engineered daily-level features derived from zero-crossing rate data, heart rate, respiratory rate, and screen usage. Specifically, we computed the sum, mean, median, minimum, maximum, and standard deviation for each data stream across a 24-hour period. All other features were handled identically to the deep learning model, following the same preprocessing pipeline and input structure.

To optimize the hyperparameters of the Random Forest classifier, we performed a randomized search using *RandomizedSearchCV* from the *scikit-learn* library. The search space included the following hyperparameters: the number of trees sampled from a uniform integer distribution between 50 and 300), the maximum depth of the trees (chosen from [5, 10, 15, None]), the number of features considered at each split (drawn from a uniform integer distribution between 2 and 12), and the minimum number of samples required to be at a leaf node (sampled from 5 to 30).

### 2.5. Model validation

Given the time-series nature of our data and the potential application of our model for JITAI, we initially considered a moving window split approach (see 2.6 Deviations from Preregistration). However, similar to findings reported in (Langener et al., 2025) we observed minimal variation in the outcome variable for most participants. Consequently, a simple baseline model would have already achieved performance significantly above chance. To enhance the real-world applicability of our model, we instead opted for a subject-wise split, ensuring that model evaluation was conducted on previously unseen participants, which would make the model more useful in real-world applications (Langener & Jacobson, 2025).

**Fig. 4.**
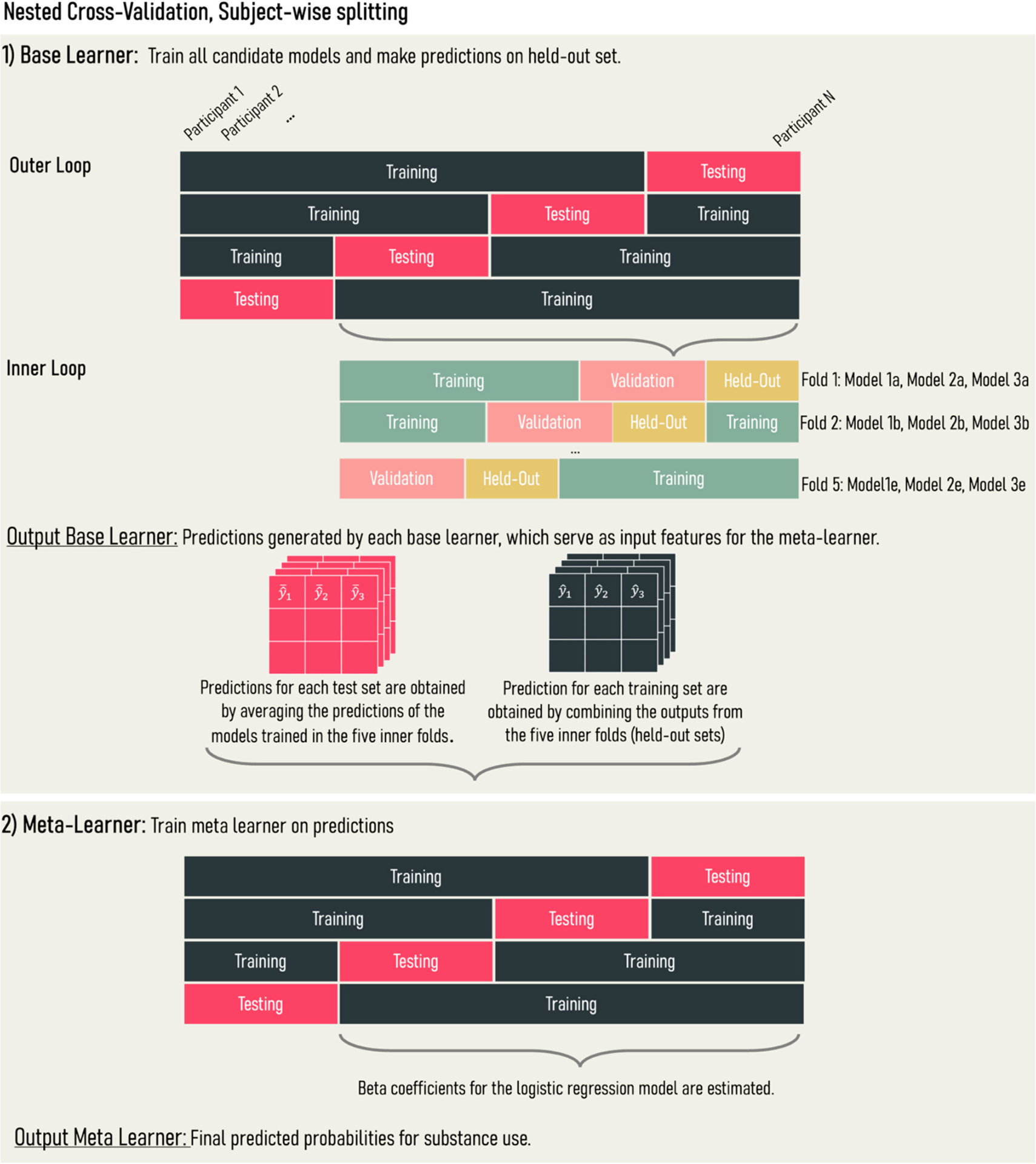
Cross Validation Split.

To evaluate model performance at the individual level for each participant, we implemented a nested cross-validation framework consisting of inner and outer loops (see Fig. 4). The base learner was first constructed, followed by the determination of the *meta*-learner.

To construct each base learner, we implemented a nested cross-validation framework using *StratifiedGroupKFold* with four outer folds. This approach ensured that data from the same participant (as identified by unique subject IDs) did not appear in both the training and testing sets within each outer fold. Due to class imbalance in our dataset (alcohol use vs. non-use), we adopted a stratified cross-validation strategy using the *scikit-learn* library (Pedregosa et al., 2011), which preserved the relative proportion of samples within each class and helped prevent biased model evaluation.

Within each outer training set, we performed a 5-fold stratified group split. One of these inner folds (20%) was held out to generate predictions for the meta learner, while the remaining 80% were used to train the base model. To enable early stopping and fine-tuning of model parameters during training, this 80% subset was further divided into a 2:1 ratio, used for training and validation.

After training, the predictions from each held-out inner fold were aggregated and used as input features for a *meta*-learner (logistic regression). For final evaluation, predictions on the original outer test set were generated by averaging the five inner-fold model predictions and feeding the result into the corresponding *meta*-learner, thereby producing ensemble predictions that leverage the strengths of each base model. This procedure was repeated across all four outer folds, yielding four separate *meta*-models.

Model performance was assessed using several metrics, including AUC, Sensitivity, Specificity, Accuracy, Kappa, and F1 Score. Given the class imbalance in our dataset, we report balanced accuracy, calculated as the average of Sensitivity and Specificity, instead of traditional accuracy. Youden’s index was applied to the training data to find the optimal probability threshold (Fluss et al., 2005). Performance was evaluated on each held-out test set (4 folds) to account for both intra- and interindividual variability. Additionally, we assessed performance within individuals (intraindividual variability). Specifically, we calculated performance metrics separately for each participant in the test set using only their own data points and then reported the average of these individual scores. Confidence intervals were computed using the bootstrapping method, implemented in the *pROC* package (1.18.5) in R (4.4.2). To assess predictive performance, we also compare our model to a baseline logistic regression model using only the day of the week as a predictor.

### 2.6. Deviations from preregistration

This paper was initially preregistered (https://osf.io/ft896) as an exploratory study; however, we made several significant deviations based on insights gained from a prior project (Langener et al., 2025).

The main change was switching from a moving window to a nested stratified k-fold cross-validation strategy, ensuring participants appear only in the training or testing set (see 2.5 Model Validation). This adjustment addressed limited within-person outcome variability. Consequently, a model trained on such data would have been ineffective in clinical practice, as a simple baseline model would have outperformed it (Langener & Jacobson, 2025), and the model would have only distinguished between individuals. Evaluating the model on unseen participants makes it more relevant for real-world clinical applications.

Additionally, we had initially planned to run separate models: one for all participants and one for those who used alcohol during the study. However, we decided to run models exclusively on participants who used alcohol, as those are the individuals who would benefit from a JITAI.

We also initially intended to predict the number of drinks per day. However, we chose to binarize this variable because the number of drinks was often missing or inconsistently reported. Participants interpreted the question differently, with responses ranging from 0.1 to 712 standard drinks in a day (M = 2.98, SD = 16.12).

We initially focused on three outcomes: 1) alcohol consumption, 2) cannabis use, 3) other substances. However, cannabis use was infrequent in our sample,^3^ and we were unable to develop a machine learning model that exceed chance level (AUC ~ 0.5), therefore, this was not pursued for this manuscript. Similarly, we did not predict other substance use due to the low variability between participants, and because the use of other substances likely included medications. Consequently, this paper only reports the results for predicting alcohol use.

A minor deviation is that we used the full sample instead of a sub-sample. This decision was made because data collection was completed by the time we began analyzing the data.

## 3. Results

### 3.1. Intra- and interindividual variability

Table 3 presents the performance metrics for alcohol detection (i.e., same-day) across the four held-out test sets, capturing both intra- and interindividual variability. The mean Sensitivity across the four folds was 0.68, indicating a moderate ability to correctly identify alcohol cases. Mean Specificity averaged 0.59, suggesting the model’s capacity to accurately identify non-alcohol cases. Mean precision/positive predictive value (ppv) was 0.24 and the mean negative predictive value (npv) was 0.91. The mean AUC was 0.67 (95% CI: 0.65–0.68) and the mean F1 Score and mean balanced accuracy were 0.35 and 0.64, respectively. The mean Cohen’s Kappa coefficient was 0.15. The mean Brier score of 0.36 indicates moderate accuracy of the predicted probabilities. Calibration analysis revealed a mean intercept of −2.46 and a mean slope of 1.13, suggesting systematic overprediction and that the predicted probabilities were too extreme (see also Fig. S1). Given these calibration limitations and the modest overall performance, clinical implementation would require substantial improvements in both accuracy and calibration. Youden’s Index was used to determine the optimal threshold for calculating Sensitivity, Specificity, Precision, Accuracy, Kappa, and F1 Score.

Importantly, when comparing the performance of our model in predicting intra- and interindividual variability to a simple baseline model that only used the weekday as a predictor, we find that our model has a slightly higher AUC (0.67 vs. 0.65) and demonstrates slightly better balanced accuracy (0.64 vs. 0.63). Additionally, it is considerably more effective at predicting alcohol use events (Sensitivity: 0.68 vs. 0.62). This higher Sensitivity results in lower Specificity compared to the baseline model (0.59 vs. 0.64). Our mean Cohen’s Kappa is slightly lower (0.16 vs. 0.16).

Thus, overall, we can see that a simple baseline model that only uses the weekday as a predictor already performs moderately well, and our model only shows slightly better performance.

Table 4 shows the model’s performance in predicting alcohol consumption (i.e., next day) on the test set, taking both intra- and interindividual variability into account. These results are based on a 4-fold cross-validation. The models exhibited performance comparable to those trained for alcohol use detection, maintaining relatively consistent results across folds. Sensitivity had an average of 0.68, reflecting a moderate ability to identify alcohol use cases correctly. Specificity, on the other hand, averaged 0.60 suggesting good performance in detecting non-alcohol use cases. Mean precision was 0.24, suggesting that only one in four positive predictions were true positives and mean npv was 0.91. The mean AUC is 0.67 (95% CI: 0.65–0.68), while the F1 score and balanced accuracy were 0.36 and 0.64, respectively. The mean Cohen’s Kappa coefficient was 0.16. A mean Brier score of 0.36 reflects moderate accuracy in the predicted probabilities. Calibration analysis revealed a mean intercept of −2.49 and a mean slope of 1.18, indicating systematic overprediction and that the predicted probabilities were too extreme (see also Fig. S2). Thus, clinical implementation would again require substantial improvements.

Similarly, when detecting alcohol use, a relatively simple baseline model already demonstrates moderate performance in predicting intra- and interindividual variability. However, our model again shows a higher ability to predict true positive cases (Sensitivity: 0.68 vs. 0.65), albeit at the cost of lower Specificity (0.59 vs. 0.62). Overall, our model achieves a similar balanced accuracy (0.64). The mean Cohen’s Kappa coefficient between both models was the same.

### 3.2. Intraindividual variability

Next, we investigate how well our model captures intraindividual variability, meaning how effectively it accounts for variability within a person. Thus, this time we evaluated the performance using test set data from each individual separately. This allows the model to be assessed based solely on its ability to capture variability within a person, independent of differences between individuals. This is particularly important for JITAI interventions, as it could provide valuable guidance on when and how to deliver an intervention. A summary of the predictive performance, averaged across all participants, is presented in Table 5.

**Fig. 5.**
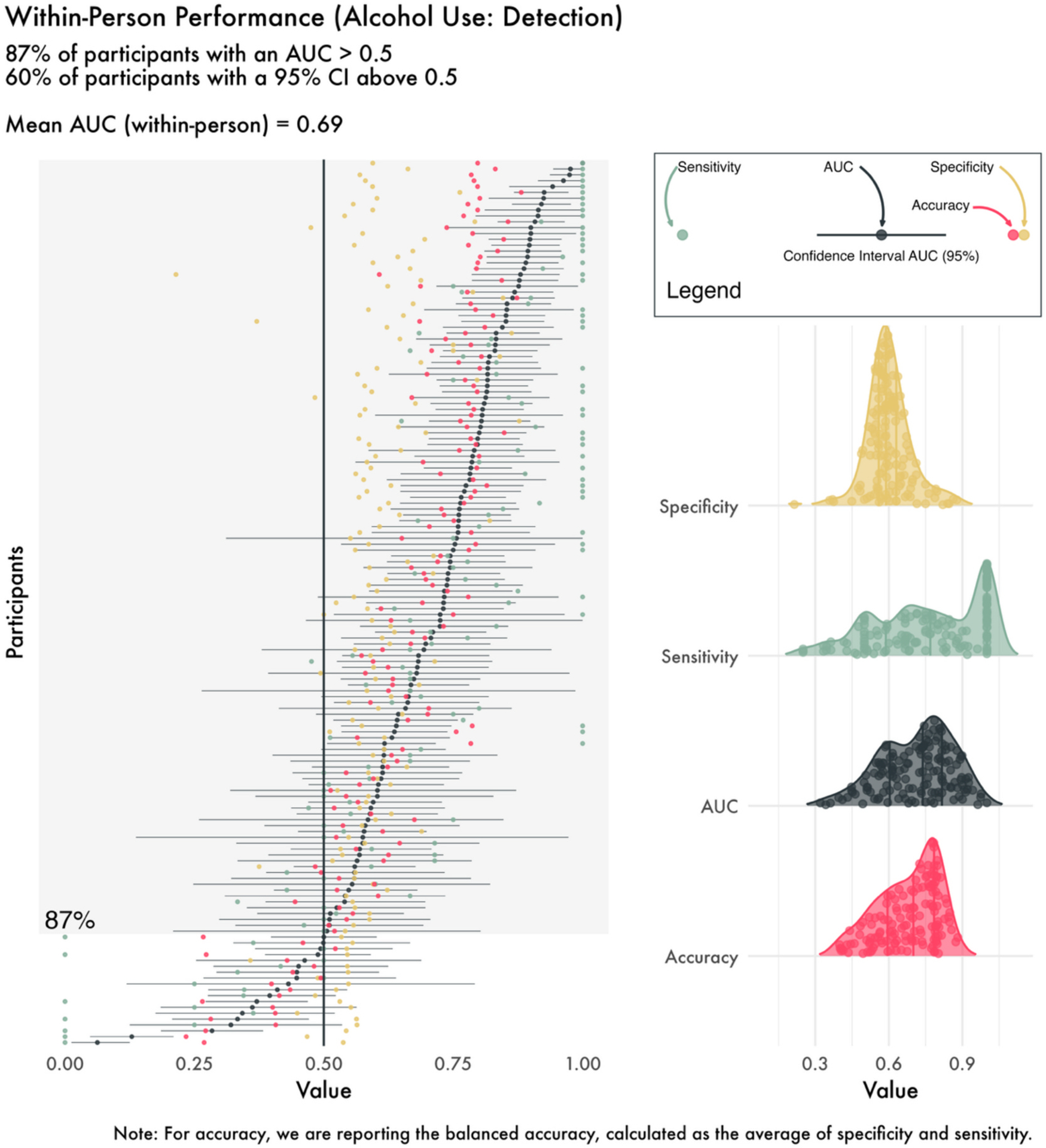
Results detecting same-day alcohol use (within-person).

Fig. 5 shows the performance for each participant. Taking the mean across all participants, Sensitivity averaged 0.72, indicating a good ability to correctly identify alcohol use cases. Specificity, on the other hand, averaged 0.60, suggesting moderate performance in detecting non-alcohol use cases. The mean AUC was 0.69. The lower bound of the CI had a mean of 0.55 (SD = 0.21), while the upper bound had a mean of 0.83 (SD = 0.15). The mean balanced accuracy was 0.66. Overall, 87.4% of participants had an AUC exceeding 0.5.

There was no significant difference in AUCs between individuals with past 12 months AUD (N = 8, M = 0.64) and individuals without past 12 months AUD (N = 151, M = 0.69), t(8.23) = −0.87, p = 0.41, 95% CI [−0.18, 0.08]. Likewise, there was no significant difference in AUCs between individuals with lifetime AUD (N = 41, M = 0.70) and individuals without lifetime AUD (N = 110, M = 0.68), t(81.18) = 0.75, p = 0.45, 95% CI [−0.04, 0.09]. Similarly, there was no significant correlation between the percentage of drinking days and the AUC (r = 0.04, t(149) = 0.454, p = 0.65).

We again compare our model’s performance to a baseline model. While the mean AUC remains the same, our model demonstrates superior performance in predicting alcohol use days (Sensitivity: 0.72 vs. 0.66). Additionally, the deep learning model results in more participants with an AUC exceeding 0.5 (87.4% vs. 83.4%).

We also investigate the model’s ability to predict alcohol drinking days within an individual (i.e., next-day drinking). Table 6 presents a summary of the results, averaged across all participants.

**Fig. 6.**
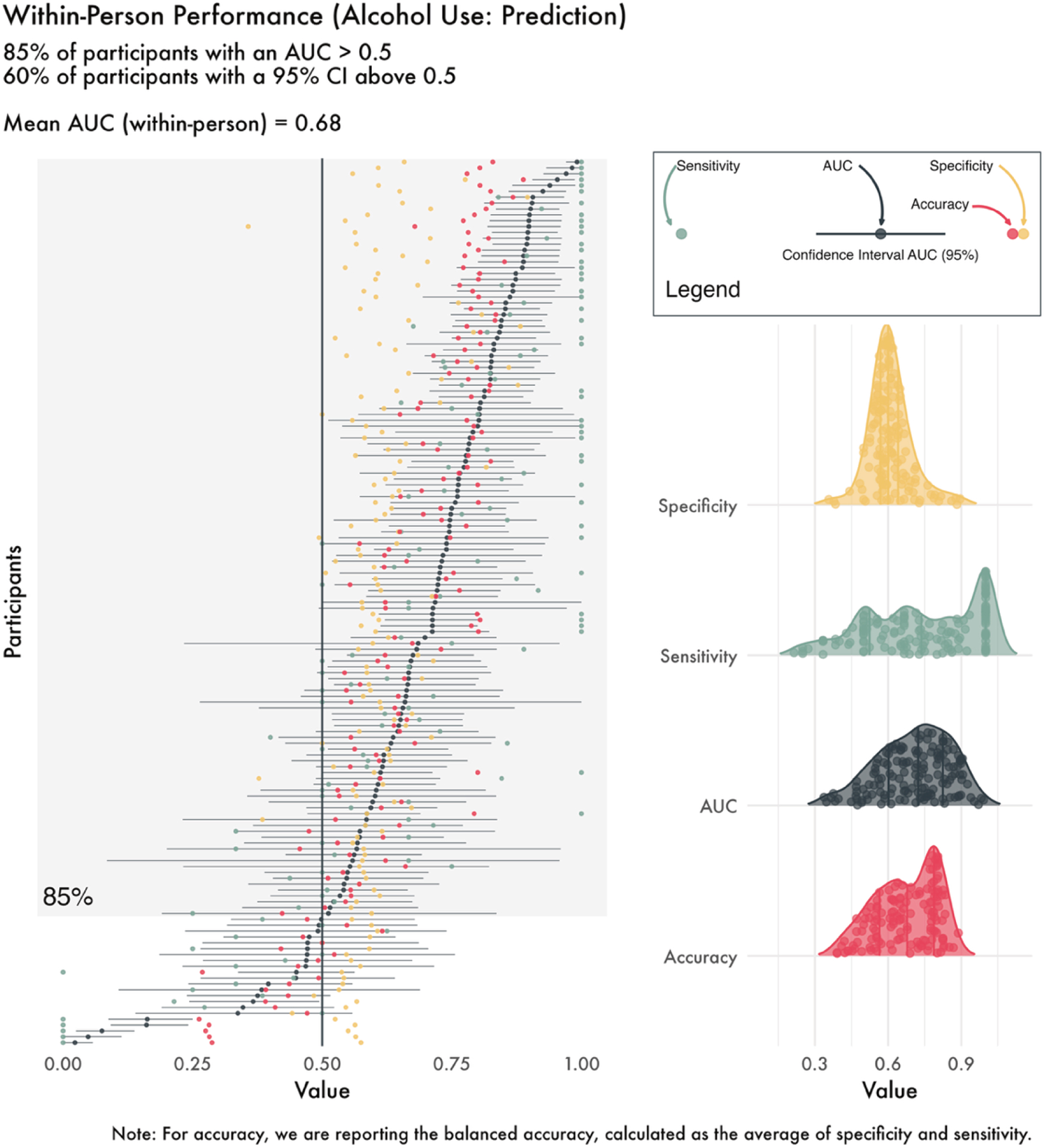
Results predicting next day alcohol use (within-person).

Fig. 6 shows the performance for each individual participant. Our model performed similarly to its performance in detecting drinking events, with a mean Sensitivity of 0.71, a mean Specificity of 0.61, and a mean AUC of 0.68. The lower bound of the CI had a mean of 0.53 (SD = 0.22), while the upper bound had a mean of 0.82 (SD = 0.17). The model had a mean Accuracy of 0.66. In total, 85.4% of participants had an AUC above 0.5. Once again, the simple baseline model showed similar overall performance in all performance measures (mean Sensitivity: 0.7, mean Specificity: 0.63, mean AUC: 0.69, mean accuracy: 0.66).

Again, there was no significant difference in AUCs between individuals with current AUD (N = 8, M = 0.63) and individuals without current AUD (N = 151, M = 0.70), t(8.07) = −1.23, p = 0.25, 95% CI [−0.19, 0.06] and no significant difference in AUCs between individuals with past 12-month AUD (N = 41, M = 0.70) and individuals without past 12-month AUD (N = 110, M = 0.69), t(73.89) = 0.23, p = 0.82, 95% CI [−0.05, 0.07]. Similarly, there was no significant correlation between the percentage of drinking days and the AUC (*r* = 0.09, *t*(149) = 1.12, *p* = 0.27).

## 4. Discussion

### 4.1. General overview

This study examined the feasibility of using deep learning models trained on passively collected smartphone and smartwatch data to detect same-day and predict next-day alcohol consumption among individuals with MDD. Specifically, we assessed whether models could generalize to new individuals, ensuring that participants were included only in either the training or testing set.

For same-day alcohol use detection, the model achieved a mean AUC of 0.67 (95% CI: 0.65–0.68). Similarly, for next-day prediction, the mean AUC was also 0.67 (95% CI: 0.65–0.68) across test folds, capturing both interindividual variability (differences between participants, such as in drinking frequency) and intraindividual variability (changes within individuals over time). While these results indicate moderate predictive performance, the model did not significantly outperform a simple baseline that used only the day of the week as a predictor (baseline AUCs: 0.65 and 0.66, respectively).

When focusing on only intraindividual prediction, tracking changes within the same person over time, the model showed similar performance. For same-day detection, the mean AUC was 0.69, and for next-day prediction 0.68. In this context, 87% and 85% of participants achieved an AUC greater than 0.5, respectively. However, this performance remained comparable to the baseline weekday-only model (AUCs: 0.68 for same-day detection and 0.69 for next-day prediction), with 83.4% and 86.8% of participants surpassing the 0.5 AUC threshold.

These findings suggest that while our deep learning models can moderately detect same-day and predict next-day alcohol use from passive sensing data, much of the predictive signal is mostly captured by simple temporal patterns, such as the day of the week, which alone serve as strong baseline predictors. Thus, our current models offer only limited advantages over a simple baseline model.

### 4.2. Implications for research and clinical practice

Our findings highlight the critical importance of evaluating predictive models against baselines to determine their added value in real-world applications (DeMasi et al., 2017). In this study, our deep learning models performed only marginally better than a straightforward baseline that relied solely on the day of the week.

From a clinical perspective, this suggests that simple temporal patterns, such as weekday effects, may already offer a practical foundation for predicting alcohol use and timing interventions. These findings align with previous research that also identified the day of the week as one of the most important predictors of alcohol consumption (Bae et al., 2018, 2023), this implies that scheduling intervention triggers based on the day and time of the week may already be an effective strategy for reducing alcohol use. Future research should explore how to integrate this temporal information into existing JITAI systems, many of which rely on self-reported data, to enhance their effectiveness and scalability (e.g., Coughlin et al., 2021; Walters et al., 2022).

The results suggest that additional factors may be necessary to enhance predictive accuracy beyond what can be captured by simple temporal cues like the day of the week. While our models were explicitly designed to generalize across individuals, our findings suggest that passively collected data alone may be insufficient for producing robust, cross-individual predictions that are better than baseline models. Clinically meaningful outcomes may instead require personalized models. This is consistent with prior research showing that individualized approaches often outperform generalized models in behavioral and mental health contexts (Abdullah et al., 2016; Benoit et al., 2020). Furthermore, hybrid approaches that integrate both individualized and non-individualized components have been effective in capturing both intraindividual and interindividual variability in mood symptoms (Jacobson et al., 2020). However, a major barrier to implementing individualized models is the need for sufficient within-person data. Despite our study’s relatively long data collection period, the volume of data available for each participant was insufficient to reliably train personalized models.

Another important consideration for clinical application is the challenge of distinguishing casual from problematic alcohol use. Even after filtering for participants with variability in drinking behavior, the majority consumed alcohol only once or twice per week and did not have current or past AUD. While this frequency may not be optimal from a health perspective, it likely reflects typical social drinking rather than clinically significant alcohol use, which does not directly impact the persistence of MDD (Schouten et al., 2023). This ambiguity complicates interpretation and decision making in when to intervene. For future research, it would be valuable to make a clear distinction between any alcohol use and alcohol use that could lead into problematic drinking, allowing for more targeted and clinically meaningful predictions.

### 4.3. Limitations

Our study has several strengths, including a rigorous nested cross-validation framework aimed at enhancing generalizability, and a comprehensive evaluation of the model’s ability to capture both intra- and interindividual variability, as well as intraindividual variability alone, in alcohol consumption. Additionally, we leveraged a large clinical sample of individuals diagnosed with MDD. However, several limitations should be considered when interpreting these findings.

While we evaluated our model’s performance in predicting intraindividual variability, it is important to note that these estimates may be unstable for some participants. Specifically, a few participants consumed alcohol infrequently, only once or twice during the study (see Fig. 2), which could result in overly optimistic performance estimates for those individuals. Although baseline characteristics such as alcohol use severity or sex could theoretically improve model performance, our approach focuses on day-level drinking data to ensure applicability to new participants without requiring additional information.

We experimented with multiple time scales for aggregating sensor data (e.g., hourly, daily), however, these explorations were not systematic or exhaustive. Additionally, we did not examine how much past data on a fine-grained temporal scale is necessary to optimize predictions, as Bae and colleagues (2021) did, because the Timeline Followback method does not provide the precise timing of drinking events. It is possible that an alternative temporal resolution or different lengths of historical data might yield better predictive performance. More systematic exploration of temporal features, their integration, and the optimal amount of past data remains an important area for future work.

Additionally, the classification of contextual information using Google Places data may have introduced errors. Based on the Google Places classification, we further classified places into substance use and non-substance use related locations. Those categories were not validated, thus, it is possible that places were misclassified. Such misclassifications could reduce the model’s ability to detect relevant behavioral patterns.

Passive sensing data were used as potential predictors of alcohol use. However, it is important to note that these measures may also be affected by other outcomes, such as depression severity. Previous studies have shown that depression is associated with lower levels of daily activity and movement (e.g., R. Wang et al., 2014). Future work could explore how fluctuations in depression severity influence the predictive performance of models for alcohol use.

One limitation of the current study is the reliance on weekly retrospective self-reports to determine daily alcohol use. Participants completed the Timeline FollowBack (Robinson et al., 2014) once per week, recalling which days they consumed alcohol in the prior seven days. Although this method is often used, it is susceptible to recall bias. This may introduce noise into the ground truth labels. Such inaccuracies could reduce the reliability of the labels used to train and evaluate the model, ultimately limiting performance.

A related limitation is the temporal mismatch between the high-resolution passive sensor data and the lower-resolution outcome data. Passive data were collected continuously at a fine-grained level, but alcohol use labels were only derived once per week, retrospectively. This creates a resolution gap, while the sensor data can capture within-day fluctuations, the labels only indicate whether alcohol was consumed on a given day, with no information about exact timing. This mismatch likely constrained the model’s ability to learn fine-grained patterns that precede drinking episodes and helps explain the moderate performance observed.

Future studies should prioritize temporally aligned outcome measures to more precisely link passive data to behavior. For example, we recommend collecting temporally aligned outcome data, for example via EMA prompts, event-based self-reports with precise timestamps, or shorter recall windows. Additionally, modeling approaches such as time-to-event or survival analyses could be used to capture risk windows rather than relying on binary day-level outcomes. These strategies would enable more accurate linking of passive sensor data to behavior and could improve detection and prediction of alcohol use in JITAIs.

### 4.4. Conclusion

This study demonstrates that deep learning models using passively collected smartphone and smartwatch data can moderately predict alcohol consumption among individuals with MDD. However, the predictive performance was comparable to a simple baseline model relying solely on the day of the week, underscoring that temporal patterns alone provide a strong predictive signal. These findings suggest that interventions timed based on day-of-week patterns may already be effective in this population. Furthermore, the results highlight the potential necessity of developing personalized models trained on richer, individual-specific data to improve prediction accuracy and clinical utility. Future progress in digital mental health likely hinges on hybrid models that can leverage population-level temporal patterns while fine-tuning predictions to the unique digital footprint of the individual.

## Supplementary Material

1

## Figures and Tables

**Table 1:** Demographics.

Variable	Value	Mean/Count	SD/%
Age	–	37.9	10.7
Gender	Female	127	83%
	Male	15	9.8%
	Non-Binary	8	5.2%
	Other	3	2%
Race	White	125	81.7%
	Hispanic/Latino	16	10.5%
	Black or African American	11	7.2%
	Multiple	6	3.9%
	Asian	5	3.3%
	Other	5	3.3%
	American Indian or Alaska Native	1	0.7%
AUD	Past Year	8	5.2%
	Lifetime	42	27.5%

**Table 2:** Overview of included features.

Sensor	Feature	Timescale for Aggregation	Type	Descriptives
Accelerometer	Mean ZCR	Per minute throughout a day	Continuous	Mean: 313.83
				Median: 338.5
				Min: 1
				Max: 712
				Std: 61.64
Heart rate	Mean heart rate	Per minute throughout a day	Continuous	Mean: 81.41
				Median: 79.8
				Min: 28
				Max: 200
				Std: 16.26
Respiratory rate	Mean respiratory rate	Per minute throughout a day	Continuous	Mean: 14.47
				Median: 14
				Min: 6
				Max: 30
				Std: 2.27
Screen Usage	Sum screen turned on	Per minute throughout a day	Continuous	Mean: 13.36
				Median: 0
				Min: 0
				Max: 60
				Std: 25.26
GPS	Visited places (substance use-related locations, liquor stores, alcohol purchasing locations, restaurants, sports, and travel)	Daily	Categorical	Substance use-related: 226
				Liquor stores, alcohol: 94
				Purchasing locations: 2028
				Restaurants: 1896
				Sports: 212
				Travel: 236
				Other:13273
GPS	Minimum distance to a cannabis dispensary	Daily	Continuous	Mean: 45.27
				Median: 2.87
				Min: 0
				Max: 5013.97
				Std: 210.45
Timestamp	Weekday	Daily	Categorical	–

**Table 3:** Detecting same-day alcohol results test set (intra- and interindividual variability).

Fold	Deep Learning Sensitivity	Specificity	AUC	Kappa	F1 Score	Accuracy	Baseline Sensitivity	Specificity	AUC	Kappa	F1 Score	Accuracy
1	0.67	0.56	0.64	0.13	0.34	0.61	0.43	0.74	0.64	0.13	0.31	0.59
2	0.75	0.60	0.70	0.17	0.35	0.67	0.72	0.61	0.69	0.18	0.35	0.67
3	0.64	0.61	0.65	0.14	0.33	0.63	0.66	0.61	0.63	0.15	0.33	0.63
4	0.68	0.59	0.67	0.17	0.4	0.63	0.65	0.62	0.65	018	0.40	0.64
Mean	0.68	0.59	0.67	0.15	0.35	0.64	0.62	0.64	0.65	0.16	0.35	0.63

*Note*. Accuracy is calculated as the balanced accuracy taking the average of sensitivity and specificity. Baseline model includes only day of week as predictor. Youden’s index was applied to the threshold related to calculating Sensitivity, Specificity, Accuracy, Kappa and F1 Score.

**Table 4:** Predicting next day alcohol results test set (intra- and interindividual variability).

Fold	Deep Learning Sensitivity	Specificity	AUC	Kappa	F1 Score	Accuracy	Baseline Sensitivity	Specificity	AUC	Kappa	F1 Score	Accuracy
1	0.67	0.59	0.67	0.16	0.37	0.63	0.59	0.63	0.64	0.14	0.35	0.61
2	0.69	0.62	0.68	0.17	0.36	0.65	0.69	0.62	0.67	0.18	0.36	0.65
3	0.77	0.59	0.71	0.2	0.39	0.68	0.72	0.63	0.70	0.21	0.39	0.68
4	0.58	0.61	0.62	0.11	0.31	0.59	0.60	0.60	0.62	0.12	0.32	0.60
Mean	0.68	0.60	0.67	0.16	0.36	0.64	0.65	0.62	0.66	0.16	0.36	0.64

*Note*. Accuracy is calculated as the balanced accuracy taking the average of sensitivity and specificity. Baseline model includes only day of week as predictor. Youden’s index was applied to the threshold related to calculating Sensitivity, Specificity, Accuracy, Kappa and F1 Score.

**Table 5:** Detecting same-day alcohol results test set (intraindividual variability).

	Mean Sensitivity	Mean Specificity	Mean AUC	Mean Accuracy	AUC < 0.5
Baseline	0.66 (± 0.29)	0.65 (± 0.09)	0.68 (± 0.18)	0.66 (± 0.15)	83.4%
Deep Learning	0.72 (± 0.27)	0.60 (± 0.09)	0.69 (± 0.17)	0.66 (± 0.15)	87.4%

*Note*. Accuracy is calculated as the balanced accuracy taking the average of sensitivity and specificity. Baseline model includes only day of week as predictor.

**Table 6:** Predicting next day alcohol results test set (intraindividual variability).

	Mean Sensitivity	Mean Specificity	Mean AUC	Mean Accuracy	AUC < 0.5
Baseline	0.70 (± 0.27)	0.63 (± 0.07)	0.69 (± 0.17)	0.66 (± 0.15)	86.8%
Deep Learning	0.71 (± 0.27)	0.61 (± 0.08)	0.68 (± 0.18)	0.66 (± 0.15)	85.4%

## Data Availability

The data will be made available as part of the NIMH data archive, but will remain under embargo until the study is completed.

## Footnotes

1 No weekly data were received for one participant.

2 We initially preregistered two primary outcomes: alcohol consumption and cannabis use (see Deviations from Preregistration). However, cannabis use was relatively infrequent in our sample, and despite our efforts, we were unable to develop a machine learning model that achieved satisfactory predictive performance for cannabis use. We include this information here for transparency. Consequently, this paper focuses exclusively on presenting results related to predicting alcohol use.

3 65 participants in our sample showed variability in their usage. These 65 participants had an average of 77.2 days of data (median = 81, SD = 10.6, min = 45, max = 89). On average, these participants consumed cannabis on 23.7 days out of 89 (median = 8, SD = 27.6, min = 1, max = 82).

## Appendix A. Supplementary data

Supplementary data to this article can be found online at https://doi.org/10.1016/j.addbeh.2026.108624.
