# Supplementary material for: Passive data do not improve prediction or detection of alcohol consumption beyond temporal patterns in major depression: A 90-day cross-validated study
Source: Addict Behav. Author manuscript; Available in PMC 2026 Jul 6. (PMC13334849; doi:10.1016/j.addbeh.2026.108624)
Supplement: 1 [file NIHMS2190195-supplement-1.docx]

**Supplementary Material**

**Comorbidities**

**Supplementary Table 1**

***Comorbidities***

| **Disorder** | **N** | **%** |
| --- | --- | --- |
| Major Depressive Disorder (lifetime) | 153 | 100.0 |
| Major Depressive Disorder (past month) | 153 | 100.0 |
| Generalized Anxiety Disorder (lifetime) | 97 | 63.4 |
| Persistent Depressive Disorder (past two years) | 96 | 62.7 |
| Persistent Depressive Disorder (prior to past two years) | 93 | 60.8 |
| Generalized Anxiety Disorder (past 6 months) | 89 | 58.2 |
| Social Anxiety Disorder (lifetime) | 86 | 56.2 |
| Social Anxiety Disorder (past 6 months) | 74 | 48.4 |
| Adult ADHD (past 6 months) | 57 | 37.3 |
| Posttraumatic Stress Disorder (lifetime) | 57 | 37.3 |
| Alcohol Use Disorder (prior to past 12 months) | 42 | 27.5 |
| Specific Phobia (lifetime) | 31 | 20.3 |
| Other Specified Trauma- and Stressor-Related Disorder (lifetime) | 27 | 17.6 |
| Posttraumatic Stress Disorder (past month) | 25 | 16.3 |
| Specific Phobia (past 6 months) | 23 | 15.0 |
| Other Specified Trauma- and Stressor-Related Disorder (past month) | 23 | 15.0 |
| Panic Disorder (lifetime) | 20 | 13.1 |
| Agoraphobia (lifetime) | 18 | 11.8 |
| Cannabis Use Disorder (prior to past 12 months) | 17 | 11.1 |
| Insomnia Disorder (past 3 months) | 14 | 9.2 |
| Binge Eating Disorder (lifetime) | 14 | 9.2 |
| Obsessive-Compulsive Disorder (lifetime) | 13 | 8.5 |
| Anorexia Nervosa (lifetime) | 13 | 8.5 |
| Other Specified Feeding or Eating Disorder (lifetime) | 12 | 7.8 |
| Agoraphobia (past 6 months) | 10 | 6.5 |
| Other Specified Anxiety Disorder (lifetime) | 10 | 6.5 |
| Premenstrual Dysphoric Disorder (past 12 months) | 9 | 5.9 |
| Other Specified Anxiety Disorder (past month) | 9 | 5.9 |
| Body Dysmorphic Disorder (lifetime) | 9 | 5.9 |
| Alcohol Use Disorder (past 12 months) | 8 | 5.2 |
| Cannabis Use Disorder (past 12 months) | 8 | 5.2 |
| Bulimia Nervosa (lifetime) | 8 | 5.2 |
| Other Specified Feeding or Eating Disorder (past month) | 8 | 5.2 |
| Stimulant/Cocaine Use Disorder (prior to past 12 months) | 7 | 4.6 |
| Panic Disorder (past month) | 7 | 4.6 |
| Body Dysmorphic Disorder (past month) | 7 | 4.6 |
| Binge Eating Disorder (past 3 months) | 6 | 3.9 |
| Obsessive-Compulsive Disorder (past month) | 5 | 3.3 |
| Other Specified OCD and Related Disorder (lifetime) | 4 | 2.6 |
| Other Specified OCD and Related Disorder (past month) | 3 | 2.0 |
| Sedative/Hypnotic/Anxiolytic Use Disorder (prior to past 12 months) | 2 | 1.3 |
| Opioid Use Disorder (prior to past 12 months) | 2 | 1.3 |
| Other Hallucinogen Use Disorder (prior to past 12 months) | 2 | 1.3 |
| Hypersomnolence Disorder (past 3 months) | 2 | 1.3 |
| Bulimia Nervosa (past 3 months) | 2 | 1.3 |
| Substance/Medication-Induced Depressive Disorder (lifetime) | 1 | 0.7 |
| Opioid Use Disorder (past 12 months) | 1 | 0.7 |
| Other Hallucinogen Use Disorder (past 12 months) | 1 | 0.7 |
| Other/Unknown Substance Use Disorder (past 12 months) | 1 | 0.7 |
| Anxiety Disorder Due to Another Medical Condition (lifetime) | 1 | 0.7 |
| Acute Stress Disorder (past month) | 1 | 0.7 |

## Model Architecture

For the first three heads we used a series of dense layers (see Supplementary Table 2 for full architecture), to learn meaningful patterns within each sensor type. The fourth head processes time-series data, specifically heart rate, ZCR, respiratory rate, and screen activity, which are summarized per minute throughout the day. Since time-series data captures temporal dependencies, we employ a 2D CNN architecture (Krizhevsky et al., 2017), a deep learning approach originally developed for image classification. To adapt CNNs for time-series processing, we transform the sequential data into 2D representations using a GAF (Wang & Oates, 2014). This method converts univariate time-series data into structured images while preserving temporal dependencies. Once the data is transformed into images, it passes through two convolutional layers, which extract spatial features from the generated images. These layers detect patterns in the temporal structure of the physiological signals. A max-pooling layer follows, reducing the dimensionality while retaining the most important features by selecting the maximum values from small patches of the image. This compressed representation is then flattened and fed into dense layers for further processing.

After each type of data is processed through its respective subnetwork, the outputs are concatenated into a single representation. This step allows the model to learn relationships between different types of data. For example, it might recognize that a high heart rate combined with specific Google Maps data and a certain weekday pattern is associated with a particular outcome. Thus, more dense layers were added. The final layer is a single-node output layer with a sigmoid activation function, producing a probability value between 0 and 1. This output represents the likelihood of a participant drinking alcohol.

To further enhance generalization, we incorporate dropout layers in each head, which randomly deactivate neurons during training, reducing dependency on specific patterns in the data. Batch normalization is applied across layers to ensure stable learning by normalizing feature distributions. Additionally, some inputs contain missing values; to address this, masking layers are used in different heads to ignore invalid or missing data points. Note that data was only labelled as missing if it was not present for a whole day.

To optimize model training, we used the "Reduce Learning Rate on Plateau" callback in *Keras*, which dynamically adjusts the learning rate when the model's performance plateaus. Specifically, we started with an initial learning rate of 0.01 and applied a decay factor of 0.7, with a patience of 20 epochs, meaning the learning rate was reduced after 20 consecutive epochs without improvement in model performance. Both the decay factor and patience were treated as hyperparameters during training, and we experimented with alternative values before selecting the final configuration. Each time the learning rate was decreased, we reverted to the best-performing model up until that point. Additionally, we utilized class weights to handle class imbalance, which were calculated using the *compute_class_weight* function from *Keras*. This helped ensure that the model treated all classes with appropriate importance. For training efficiency, we set a large batch size of 2048, enabling stable model updates.

**Supplementary Table 2**

*Full Model Architecture.*

| Head | Layer Type | Additional Information |
| --- | --- | --- |
| Google Maps | Input | - |
|  | Dense | Units: 64, Activation: ReLU |
|  | Dropout | Rate: 0.3 |
|  | Dense | Units: 32, Activation: ReLU |
|  | Dense | Units: 1, Activation: ReLU |
| Heart Rate/ ZCR/ Screen/ Respiratory Rate | Input | - |
|  | Conv2D | Filters: 96, Kernel size: (6,6), Strides: (1,1), Activation: ReLU, Kernel initializer: 'he_normal' |
|  | MaxPooling2D | Pool size: (3,3), Strides: (2,2) |
|  | Conv2D | Filters: 256, Kernel size: (5,5), Strides: (1,1), Activation: ReLU, Padding: 'same' Kernel initializer: 'he_normal' |
|  | BatchNormalization | - |
|  | Dropout | Rate: 0.3 |
|  | Flatten | Shape: (None, 256) |
|  | Dense | Units: 1, Activation: ReLU |
| Cannabis | Input | - |
|  | Masking | - |
|  | Dense | Units: 128, Activation: ReLU, Kernel initializer: 'he_normal' |
|  | Dropout | Rate: 0.3 |
|  | Dense | Units: 64, Activation: ReLU, Kernel initializer: 'he_normal' |
|  | BatchNormalization | - |
|  | Flatten | - |
| Weekday | Input | - |
|  | Dense | Units: 64, Activation: ReLU |
|  | Dropout | Rate: 0.3 |
|  | Dense | Units: 32, Activation: ReLU |
|  | BatchNormalization | - |
| Merged | Concatenate | *Merging outputs from all four heads* |
|  | Dense | Units: 128, Activation: ReLU, Kernel initializer: 'he_normal' |
|  | Dropout | Rate: 0.3 |
|  | Dense | Units: 64, Activation: ReLU, Kernel initializer: 'he_normal' |
|  | Dense | Units: 32, Activation: ReLU, Kernel initializer: 'he_normal' |
|  | Dropout | Rate: 0.3 |
| Output | Dense | Units: 1, Activation: Sigmoid, Kernel initializer: 'glorot_normal' |

## Model Development

The development of our final model was an iterative process that involved extensive hyperparameter tuning and the evaluation of various deep learning architectures using our validation sets. In this section, we detail some of the approaches that were tested but ultimately did not result in improved model performance, and therefore were excluded from the final architecture.

One of the initial adjustments we explored was the inclusion of features on different time scales. For example, we initially tested using the visited places data at a finer, minute-by-minute level. However, we found that a daily time scale often yielded better results. This may be because the daily time scale aligns more closely with the outcome variable, although this observation requires further investigation as the tests were not systematically conducted across all models. In addition, we also experimented with Long Short-Term Memory (LSTM) layers for the Google Maps and cannabis dispensary features to capture temporal relationships. However, this did not lead to better performance either, suggesting that the additional complexity introduced by LSTMs did not provide significant benefits in this context.

For the cannabis dispensary data, we experimented with varying the features, such as testing different distances for proximity (e.g., dispensaries within a 1-mile radius versus a 5-mile radius). Despite these variations, no substantial improvements in model performance were observed, suggesting that the minimum distance captured the most relevant information.

We also tested the inclusion of raw, scaled latitude and longitude data summarized for each minute as additional predictors. Although similar features have been successful in previous studies (Bae et al., 2023), this approach seemed to worsen our model’s predictions, indicating that the high-resolution spatial data did not offer useful information for the prediction task at hand.

We also explored different configurations of regularization techniques, particularly by adjusting the dropout rate. Similarly, we experimented with different sizes for the dense layers within the architecture. We also experimented with different batch sizes and starting learning rates as well as the decay factor of the learning rate, but our final configurations performed best on the validation set.

Given that more complex meta-learners like XGBoost offered no performance gains, a logistic regression model was chosen for its simplicity and interpretability.

**Random Forest Model Results**

**Supplementary Table 2**

*Random Forest Results*

|  | Detect | | | | | | Predict | | | | | |
| --- | --- | --- | --- | --- | --- | --- | --- | --- | --- | --- | --- | --- |
| Fold | Sensitivity | Specificity | AUC | Kappa | F1 Score | Accuracy | Sensitivity | Specificity | AUC | Kappa | F1 Score | Accuracy |
| 1 | 0.76 | 0.47 | 0.64 | 0.11 | 0.34 | 0.62 | 0.67 | 0.61 | 0.69 | 0.17 | 0.36 | 0.64 |
| 2 | 0.78 | 0.47 | 0.66 | 0.11 | 0.32 | 0.63 | 0.46 | 0.71 | 0.62 | 0.12 | 0.30 | 0.59 |
| 3 | 0.69 | 0.57 | 0.65 | 0.16 | 0.39 | 0.63 | 0.60 | 0.61 | 0.64 | 0.13 | 0.35 | 0.61 |
| 4 | 0.63 | 0.63 | 0.66 | 0.14 | 0.31 | 0.63 | 0.68 | 0.56 | 0.65 | 0.13 | 0.55 | 0.62 |
| Mean | 0.71 | 0.53 | 0.65 | 0.13 | 0.34 | 0.62 | 0.61 | 0.63 | 0.65 | 0.14 | 0.34 | 0.62 |

*Note.* Accuracy is calculated as the balanced accuracy taking the average of sensitivity and specificity. Baseline model includes only day of week as predictor. Youden’s index was applied to the threshold related to calculating Sensitivity, Specificity, Accuracy, Kappa and F1 Score.

**Calibration Curves**

For the detection task, the model appears to overpredict since the points are mostly below the diagonal line (Figure S1), indicating that the actual probabilities are generally lower than the model’s predicted probabilities. This pattern is also reflected in the calibration intercept and slope, which quantify the degree of miscalibration.

**Figure S1**

*Calibration Curve Detection*

**
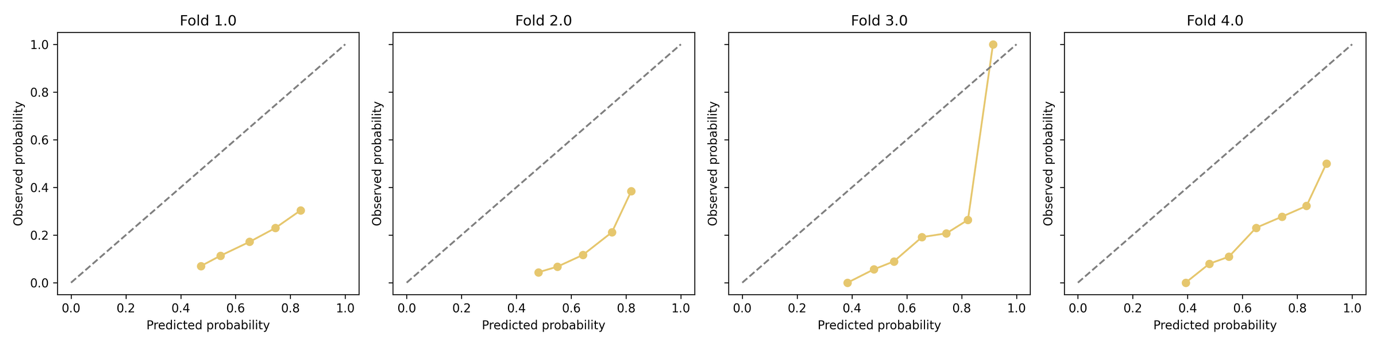
**

For the prediction task, we observe the similar pattern; the model appears to overpredict since the points are also mostly below the diagonal line (Figure S2).

**Figure S2**

*Calibration Curve Prediction*


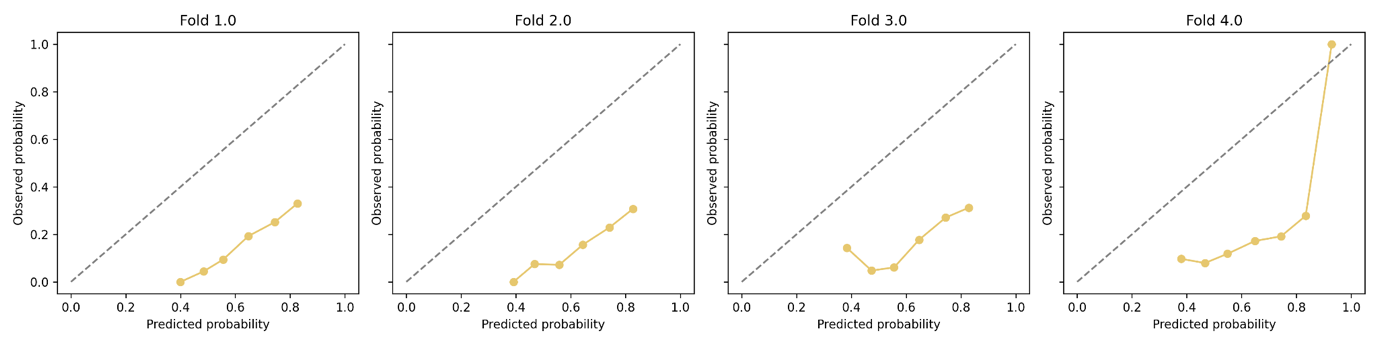


# References

Bae, S. W., Suffoletto, B., Zhang, T., Chung, T., Ozolcer, M., Islam, M. R., & Dey, A. K. (2023). Leveraging Mobile Phone Sensors, Machine Learning, and Explainable Artificial Intelligence to Predict Imminent Same-Day Binge-drinking Events to Support Just-in-time Adaptive Interventions: Algorithm Development and Validation Study. *JMIR Formative Research*, *7*(1), e39862. https://doi.org/10.2196/39862

Krizhevsky, A., Sutskever, I., & Hinton, G. E. (2017). ImageNet classification with deep convolutional neural networks. *Commun. ACM*, *60*(6), 84–90. https://doi.org/10.1145/3065386

Wang, Z., & Oates, T. (2014). *Encoding Time Series as Images for Visual Inspection and Classification Using Tiled Convolutional Neural Networks*. https://www.semanticscholar.org/paper/Encoding-Time-Series-as-Images-for-Visual-and-Using-Wang-Oates/e90666552aaaa056bc6465019632bf06917c842c
